# Independent Testing of Published CT Models for PD-L1 Status in Non–Small Cell Lung Cancer

**DOI:** 10.1148/radiol.241962

**Published:** 2026-03-31

**Authors:** Robert O’Shea, Carolyn Horst, Thubeena Manickavasagar, Eleni Josephides, Sarah Hunter, Phillipe Taniere, Daisuke Nonaka, Mieke Van Hemelrijck, James Spicer, Daniel J Hughes, Gary J. R. Cook, Andrea Bille, Eleni Karapanagiotou, Vicky Goh

**Affiliations:** ^1^Department of Cancer Imaging, School of Biomedical Engineering and Imaging Sciences, King’s College London, Floor 5, Becket House, 1 Lambeth Palace Rd, London SE1 7EU, UK; ^2^Department of Radiology, Guy’s and St. Thomas’ NHS Foundation Trust, London, UK; ^3^Guy’s Cancer Centre, Guy’s and St Thomas’ NHS Foundation Trust, London, UK; ^4^Department of Histopathology, University Hospitals Birmingham NHS Foundation Trust, Birmingham, UK; ^5^Department of Histopathology, Guy’s and St Thomas’ NHS Foundation Trust, London, UK; ^6^School of Cancer and Pharmaceutical Sciences, King’s College London, London, UK; ^7^King’s College London & Guy’s and St. Thomas’ PET Centre, London, UK

## Abstract

**Background:**

With the shift toward perioperative programmed cell death protein-1 and programmed cell death ligand-1 (PD-L1) immunotherapy in non–small cell lung cancer (NSCLC), there is a need to assess PD-L1 status preoperatively. Identifying patients who may benefit from immunotherapy using CT-based features has been hampered by the lack of independent testing.

**Purpose:**

To evaluate the performance of published CT models in predicting PD-L1 status in a multi-institutional external test set of patients with NSCLC undergoing surgery.

**Materials and Methods:**

In this retrospective study, published CT radiomic models predicting PD-L1 expression were identified by literature review spanning January 2017 to July 2023. Models with sufficient reporting quality were recreated for testing, using the features and coefficients that were originally published. Feature standardization parameters were trained in a training set, the publicly available NSCLC Cancer Imaging Archive dataset, containing images collected between April 2008 and September 2012, without label observation, for the sole purpose of test set preprocessing. For comparison, one previously published model was also retrained to predict *CD274* expression on the training set. CT model discrimination of PD-L1 tumor proportion score (TPS) at clinical thresholds of at least 1% (TPS_≥1%_) and at least 50% (TPS_≥50%_) was tested in an external test set of patients with stage IIB–IIIB NSCLC from 35 institutions studied between February 2009 and October 2018 using area under the receiver operating characteristic curve (AUC) analysis.

**Results:**

A total of 319 patients with NSCLC were included in this study (mean age, 69 years ± 8.9 [SD]; 195 male patients). Of the 17 CT radiomic models identified by literature review, only three (18%) could be reconstructed from published information (models 1–3). In the external test set (*n* = 225), model 3 demonstrated comparable TPS_≥50%_ discrimination (AUC, 0.61 [95% CI: 0.49, 0.72]; *P* = .03 [vs null]) with previously reported performance (AUC, 0.66 [95% CI: 0.58, 0.74]; *P* < .001 [test vs published]). For model 1, test TPS_≥50%_ discrimination (AUC, 0.52 [95% CI: 0.39, 0.65]; *P* = .37 [vs null]) was lower than published performance (AUC, 0.79 [95% CI: 0.58, 1.00]; *P* < .001 [test vs published]). Model 2 test TPS_≥1%_ discrimination (AUC, 0.57 [95% CI: 0.49, 0.64]; *P* = .04 [vs null]) was also lower than the published result (AUC, 0.85; *P* < .001 [test vs published]). The predictions of the *CD274* messenger RNA–fitted CT model (model 3a) correlated with PD-L1 TPS (Spearman ρ, 0.20; *P* = .001), discriminating both TPS_≥1%_ (AUC, 0.61 [95% CI: 0.55, 0.69]; *P* = .001 [vs null]) and TPS_≥50%_ thresholds (AUC, 0.66 [95% CI: 0.55, 0.76]; *P* = .001 [vs null]).

**Conclusion:**

In independent testing, CT predictive models discriminated PD-L1 expression in patients with resectable NSCLC at clinically relevant thresholds, but predictive performance was lower than initially published.

© The Author(s) 2026. Published by the Radiological Society of North America under a CC BY 4.0 license.

*Supplemental material is available for this article.*

## Graphical abstract

**Figure:**
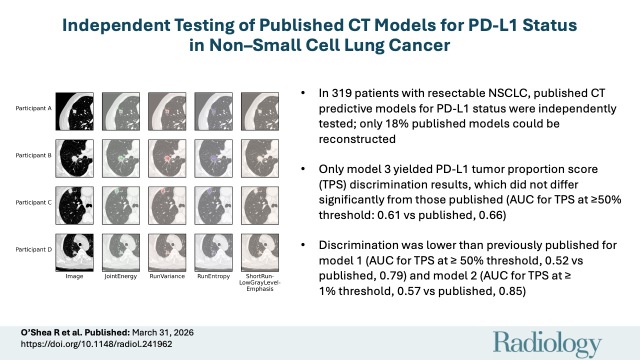

**Summary**

In independent testing, CT predictive models discriminated programmed death ligand-1 expression in patients with resectable non–small cell lung cancer at clinically relevant thresholds, but predictive performance was lower than initially published.

**Key Results**

■ In a retrospective study of 319 patients with resectable non–small cell lung cancer, published CT predictive models for programmed death ligand-1 (PD-L1) status were independently tested using a multi-institution external test set, and only three of 17 (18%) published models could be reconstructed.
■ Only model 3 yielded PD-L1 tumor proportion score (TPS) discrimination results, which did not differ significantly from those published (area under the receiver operating characteristic curve [AUC] for the TPS at a clinical threshold of at least 50% [TPS_≥50%_], 0.61 vs previously published, 0.66; *P* = .38 [testing vs published]).
■ Discrimination against PD-L1 was lower than previously published for model 1 (AUC for TPS_≥50%_, 0.52 vs published 0.79; *P* < .001 [testing vs published]) and model 2 (AUC for TPS at a clinical threshold of at least 1%, 0.57 vs published 0.85; *P* < .001 [testing vs published]).

## Introduction

Immunotherapy targeting programmed cell death protein-1 and programmed cell death ligand-1 (PD-L1) has changed the treatment landscape of advanced and/or metastatic non–small cell lung cancer (NSCLC) and improved survival (1–8). Identifying patients who are most likely to benefit from immunotherapy remains challenging due to treatment toxicity and variable response among PD-L1–positive tumors. Currently, pathologic confirmation of tumor PD-L1 protein expression is the mainstay for selecting patients for PD-L1 and/or programmed cell death protein-1 immune checkpoint inhibitor therapy. PD-L1 tumor proportion score (TPS) measured by immunohistochemistry is a validated marker (2,9). For example, in advanced NSCLC, a PD-L1 TPS at a clinical threshold of at least 50% (TPS_≥50%_) indicates a likely response to PD-L1 and programmed cell death protein-1 therapy (8). Nevertheless, pathologic assessment has limitations, including tumor heterogeneity in protein expression (10), temporal changes with treatment (11), and assay variability (12).

The phase 3 CheckMate 816 trial of nivolumab plus chemotherapy versus chemotherapy alone in the neoadjuvant setting of stage 1B–IIIA NSCLC has shown a greater event-free survival in PD-L1 TPS at a clinical threshold of at least 1% (TPS_≥1%_) (13). The phase 3 KEYNOTE-671 trial assessed whether perioperative treatment with neoadjuvant pembrolizumab plus cisplatin-based chemotherapy, surgery, and adjuvant pembrolizumab would improve the outcome compared with neoadjuvant cisplatin-based chemotherapy and surgery alone in stage II to IIIB NSCLC with TPS_≥50%_. This trial also showed higher 24-month event-free survival in the pembrolizumab versus control group (62% vs 41%) with a hazard ratio of 0.58 for progression, recurrence, or death (14).

With this shift toward perioperative use of immune checkpoint inhibitors in NSCLC (14,15), assessing PD-L1 expression at an earlier timepoint (preoperatively) will be needed. Here, imaging may complement tumor biopsy assessment. Initial CT studies suggest associations of CT imaging features with tumor PD-L1 expression and behavior, including tumor shape, and textures quantified using semiautomated or automated approaches (16,17). CT imaging models merit further exploration, but lack of independent testing and limited exploration biologic explainability has hindered clinical implementation.

This retrospective study aimed to evaluate the performance of published CT models in predicting PD-L1 status in a multi-institutional external test set of patients with NSCLC undergoing surgery.

## Materials and Methods

### Study Design

**Figure 1:**
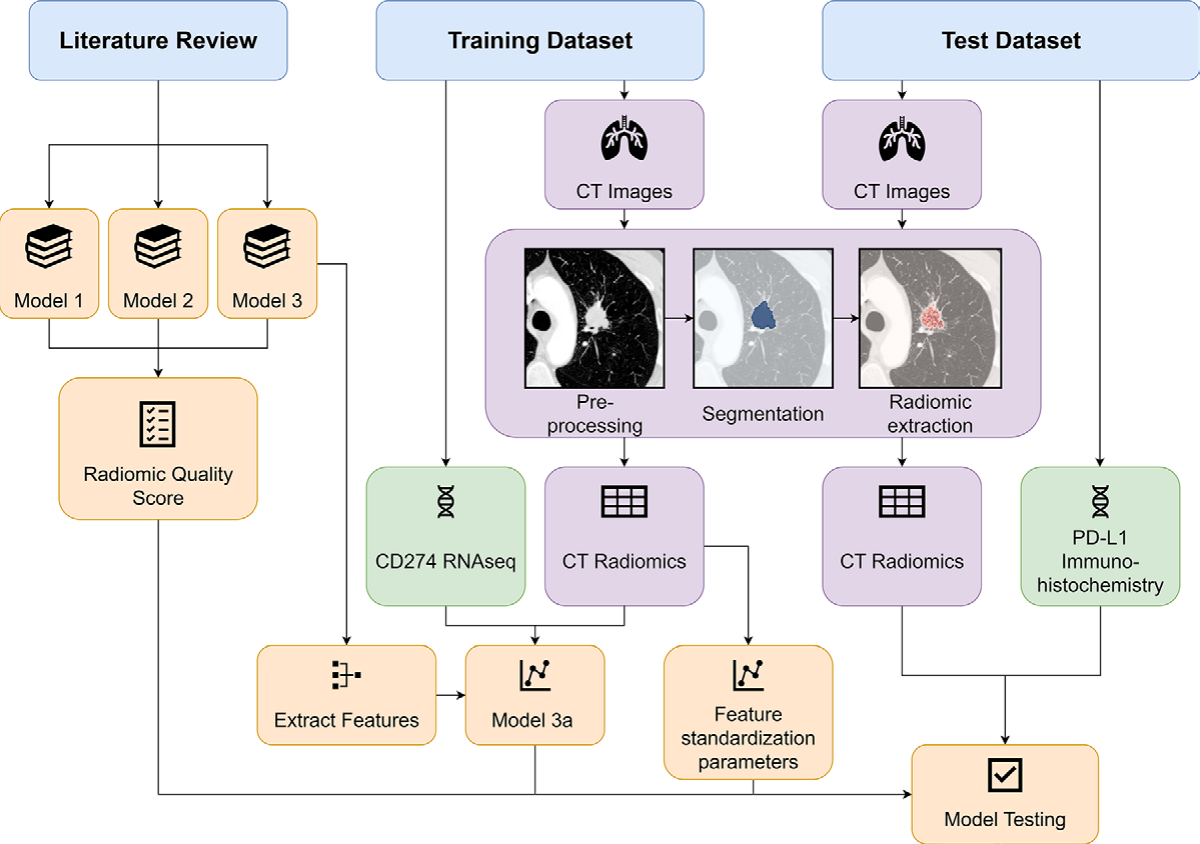
Flowchart shows study schema. Published CT radiomic models for predicting programmed death ligand-1 (PD-L1) expression were identified by literature review, and all sufficiently reported models were reconstructed for testing. Feature standardization parameters were trained on the training set, and models 1–3 were evaluated on the external test set using only the published features and coefficients. For comparison, model 3a was trained to predict *CD274* expression in the training set using the features of model 3. RNAseq = RNA sequencing.

This retrospective study was performed between July 2023 and July 2024 (18), using an external test set of imaging data collected between February 2009 and October 2018. Institutional review board approval was obtained for this retrospective analysis with opt-out consent (ethics number: 18/NW/0297) (19). The overall study schema is shown in Figure 1.

**Figure 2:**
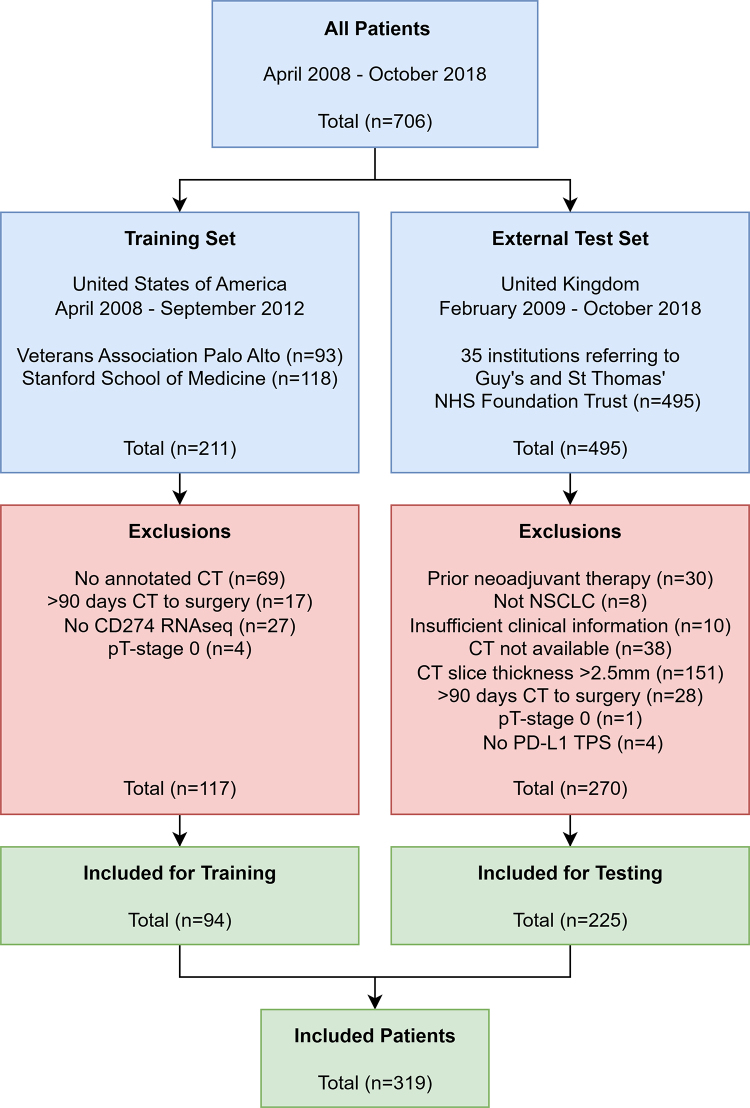
Flowchart shows patient inclusions and exclusions in the training set used for model development and the multi-institution external test set used for model testing. A total of 94 patients were included for training, whereas 225 patients were included for testing. NHS = National Health Service, NSCLC = non–small cell lung cancer, PD-L1 = programmed death ligand-1, pT-stage = pathologic tumor stage, RNAseq = RNA sequencing, TPS = tumor proportion score.

Patients were eligible for inclusion in this study if they had histologically confirmed NSCLC, with baseline CT imaging and either PD-L1 immunohistochemistry or *CD274* gene expression from tumor biopsy (Fig 2). Patients were excluded if *(a)* CT image or annotation was unavailable, *(b)* baseline CT preceded surgery by more than 90 days, *(c)* CT section thickness was more than 2.5 mm, *(d)* neither *CD274* RNA sequencing nor PD-L1 TPS was available, *(e)* neoadjuvant chemotherapy or chemoradiotherapy was administered before CT imaging, or *(f)* clinical information was insufficient. CT imaging acquisition parameters for both datasets are provided in Table S1 (mean tube current, 291 mA ± 165 [SD]; mean peak kilovoltage, 117 ± 6.7; mean voxel width, 0.75 mm ± 0.09).

### Training Set

The training set comprised the Cancer Imaging Archive NSCLC radiogenomic dataset (*n* = 211) (20) including operable NSCLC cases from Stanford University School of Medicine and the Palo Alto Veterans Affairs Healthcare System between April 2008 and September 2012 (21,22). It contained clinical data, CT, and fluorine 18 (^18^F) fluorodeoxyglucose (FDG) PET/CT images with tumor segmentations, plus gene expression and RNA sequencing data from surgically excised tumors. *CD274* is a protein-encoding gene for PD-L1.

### External Test Set

The external test set (*n* = 495) included adult patients with pathologically confirmed stage IIB–IIIB primary NSCLC who were referred from 35 institutions for primary resection and lymphadenectomy at a tertiary thoracic surgery center (Guy’s and St Thomas’ NHS Foundation Trust, London, UK) between February 2009 and October 2018, undergoing contrast-enhanced CT and ^18^F-FDG PET/CT. The ^18^F-FDG PET data from a subset of this patient sample have been published previously (*n* = 108) (23). Training and test sets were mutually exclusive in terms of institutions and patient data. Patients were excluded from the external test set if *(a)* baseline CT imaging from the referring institution was unavailable, *(b)* CT preceded surgery by more than 90 days, *(c)* CT section thickness more than 2.5 mm, *(d)* no NSCLC at pathologic examination, *(e)* PD-L1 immunohistochemistry was unavailable, *(f)* prior neoadjuvant chemotherapy or chemoradiotherapy administered, or *(g)* insufficient clinical information.

### Literature Review and Model Search

**Figure 3:**
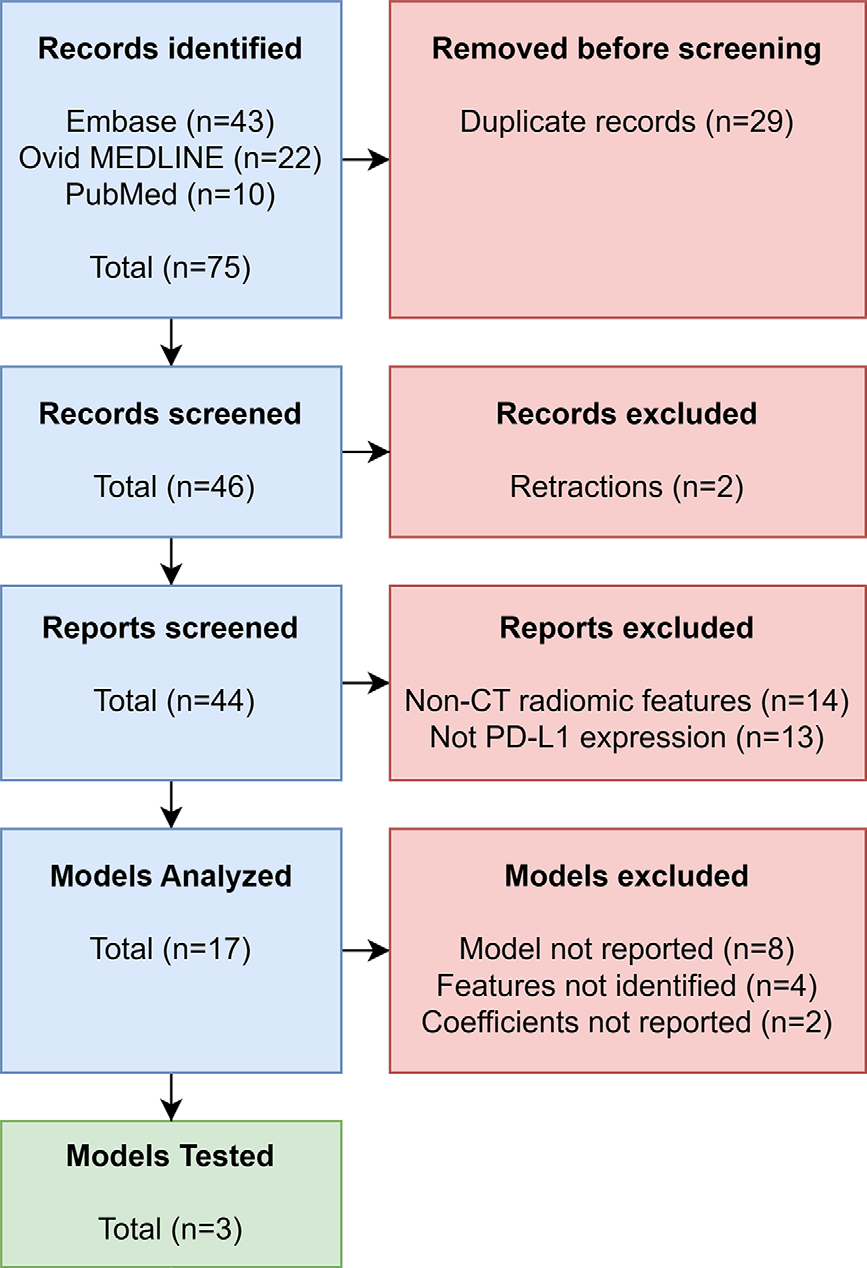
Flowchart shows Preferred Reporting Items for Systematic reviews and Meta-Analyses of literature screening. Three models were included for testing. PD-L1 = programmed death ligand-1.

A literature review was performed to identify published CT models predicting PD-L1 expression in NSCLC. PubMed, EMBASE, and MEDLINE databases were searched systematically between January 2017 and July 2023. Inclusion criteria were *(a)* original research modeling CT radiomics to predict PD-L1 expression in human NSCLC and *(b)* English language. Exclusion criteria were *(a)* prediction of outcomes other than expression (eg, therapeutic response or survival), *(b)* use of nonradiomics imaging features (eg, convolutional neural networks), and *(c)* models not adequately reported, lacking either source code or a complete list of features and coefficients (Fig 3). The search syntax for PubMed and Ovid database portals is provided in Appendix S1. Screening for inclusion was performed by three independent readers (R.O., C.H., and T.M., with 3, 5, and 2 years of experience, respectively); disagreements were resolved by consensus, with arbitration from a senior reviewer (V.G.). Articles from a previous review of radiomics models for NSCLC in PD-L1 were also included.

### Assessment of Model Reconstructibility

Published models were assessed for reconstructibility based on the following: *(a)* radiomic features were identifiable within the Image Biomarker Standardization Initiative framework (24) and *(b)* availability of model parameters (eg, coefficients, thresholds) in article text or source code. Reconstructed models were tested in the external test set of resected NSCLC without retraining. In a secondary analysis, the highest quality model according to the radiomic quality score (25) was also retrained to predict *CD274* (a protein-encoding gene for PD-L1) messenger RNA (mRNA) expression in the training set (20).

### Assessment of Model Reproducibility

Studies were analyzed by a clinical research fellow with 4 years of experience in machine learning and radiomics. Models whose features and parameters could be reconstructed from reported data or source code were included for testing. Study authors were not contacted for model information. Reproducibility was evaluated using the following criteria: *(a)* features identifiable within the International Standardization of Biomarkers Initiative taxonomy, *(b)* linear models with a small number of features, *(c)* use of publicly available radiomics libraries, *(d)* model testing in external datasets, and *(e)* radiomics quality score value.

### CT Image Analysis

**Tumor segmentation**

The training set provided CT segmentation maps. For the external test set, the primary tumor visualized at CT was segmented in two ways: first by a radiologist in training (6 years of experience) and an oncology resident (3 years of experience) in consensus using ITK-snap (version 0.1.1; *www.itksnap.org*) (26) in consultation with CT reports and staging ^18^F-FDG PET/CT. Uncertain tumor boundaries were resolved by a senior radiologist (>20 years’ experience). In a secondary analysis, a deep learning segmentation model was trained on the training set and deployed on the external test set as an alternative method; architecture and training details are provided in Appendix S1.

**Extraction of tumor CT features**

Voxel attenuation arrays were extracted from CT Digital Imaging and Communications in Medicine (DICOM) files and converted to Hounsfield units using PyDICOM, applying a 100 HU lower bound to exclude air from segmentation masks. Tumor radiomics were extracted with PyRadiomics version 3.0.1 (27), reproducing published extraction parameters where available; otherwise Ligero and colleagues’ (28,29) recommendations were used (no normalization, 10 HU fixed width bins; spline interpolation to 1 mm^3^).

### Immunohistochemical Analysis of PD-L1 Protein Expression

Following surgery, immunohistochemistry for PD-L1 protein expression was performed for the primary tumor using the clone 22C3 pharmDx assay on the Dako Automated Link 48 platform (Agilent Technologies) in an accredited reference laboratory (30). A minimum of 100 viable tumor cells were required for analysis, and stained slides were assessed according to the manufacturer’s guidelines by two independent histopathologists (each with >15 years of experience). PD-L1 TPS was the percentage of viable cancer cells with complete or partial membrane staining. Necrotic areas were excluded from scoring. Discrepant results were reassessed by the two histopathologists jointly, generating a single consensus score. PD-L1 TPS ground truth was classified as: less than 1% (no expression), 1%–49% (weak expression), and at least 50% (strong expression). TPS categories reflect clinical practice, using 1% and 50% thresholds, respectively, for management decisions in early- and advanced-stage disease.

### Statistical Analysis

Differences in patient characteristics across datasets were tested using the Fisher exact test for categorical variables and an unpaired two-sided *t* test for age. Analyses were performed by an author (R.O.S.) in R (version 4.1.1; R Foundation for Statistical Computing). For comparability, all *P* values are unadjusted. Significance threshold was α = .05.

### Model Training and Testing

In the primary analysis, all reconstructed models were tested without training, using only published information. In a secondary analysis, the highest radiomic quality score was refitted on the training set, using *CD274* expression as a surrogate label. To prevent data leakage, feature standardization parameters were derived from the training set and applied to the external test set. Where preprocessing details were unreported, models were tested with and without standardization. Parameters are summarized in Table S2.

### Model Discrimination

TPS discrimination was assessed using a one-sided Spearman test. Thresholds TPS_≥1%_ and TPS_≥50%_ were evaluated with the area under the receiver operating characteristic curve (AUC) and tested by means of comparison with an invariant predictor with a one-sided DeLong test. Otherwise, metric distributions were compared with bootstrap-based two-sample tests. Where model intercept and threshold were available, calibration was measured with accuracy, sensitivity and specificity, and χ^2^ goodness-of-fit. The 95% CIs were derived from 2.5th and 97.5th percentiles of 1000 replacement bootstrap resamples. For interpretability, *P* values for metric significance were not adjusted for multiple testing.

### Additional Model Fitting

The most reproducible CT imaging model was fitted using unpenalized linear regression for predicting *CD274* mRNA expression quantile in the training set. Features were standardized to zero mean and unit variance before model fitting using parameters from the training set. Model parameters are summarized in Table S2.

### Post Hoc Analyses

Partial correlation analysis was performed to aid model interpretation and assess volume confounding. A partial correlation matrix was computed including *CD274* quantile, PD-L1 TPS quantile, tumor volume, and the imaging predictors in the optimal model. The 95% CIs were computed by bootstrapping.

To assess segmentation impact on PD-L1 prediction, models were applied to features from deep learning–generated tumor masks, including only cases with a Dice score of more than 0.5 versus manual annotation. A second analysis excluded tumor volumes of less than 5 mL.

Image processing libraries are detailed in Appendix S1. Code required for segmentation modeling, radiomic extraction, and analysis is provided at *https://github.com/robertoshea/nsclc_pdl1_radiomics*.

## Results

### Patient Characteristics

This study included 319 patients (mean age, 69 years ± 8.9 [SD]; age range, 40–89 years; 195 male patients; 94 patients in the training set, 225 in the external test set) (Table 1). Of the initial 211 patients in the training set, 117 patients were excluded (no annotated CT, 69 patients; >90 days to surgery, 17 patients; no *CD274* RNA sequencing, 27 patients; pathologic tumor stage 0, four patients), leaving 94 patients for model development (Fig 1). Of the initial 495 patients in the external test set, 270 were excluded (prior neoadjuvant therapy, 30 patients; not NSCLC, eight patients; insufficient clinical information, 10 patients; CT unavailable, 38 patients; CT section thickness >2.5 mm, 151 patients; >90 days CT to surgery, 28 patients; pathologic tumor stage 0, one patient; no PD-L1 TPS, four patients), leaving 225 patients for model testing. Tumor segmentation was reviewed and finalized by consensus in 16 of the 225 patients in the external test set.

**Table 1:** Patient Characteristics

Characteristic	All Patients (*n* = 319)	Training Set (*n* = 94)	External Test Set (*n* = 225)	*P* Value
Age (y)*	69 ± 9	69 ± 9	69 ± 9	.89
Sex				.008
Male	195 (61.1)	68 (72.3)	127 (56.4)	
Female	124 (38.9)	26 (27.7)	98 (43.6)	
History of tobacco use				<.001
No	28 (8.78)	14 (14.9)	14 (6.22)	
Yes	262 (82.1)	80 (85.1)	182 (80.9)	
Unknown	29 (9.09)	0 (0)	29 (12.9)	
Pathologic tumor stage				<.001
T1	91 (28.5)	43 (45.7)	48 (21.3)	
T2	155 (48.6)	37 (39.4)	118 (52.4)	
T3	58 (18.2)	10 (10.6)	48 (21.3)	
T4	15 (4.70)	4 (4.26)	11 (4.89)	
Pathologic nodal stage				<.001
N0	74 (23.2)	74 (78.7)	0 (0)	
N1	129 (40.4)	8 (8.51)	121 (53.8)	
N2	116 (36.4)	12 (12.8)	104 (46.2)	
PD-L1 TPS				<.001
<1% (negative)	NR	23 (24.5)	124 (55.1)	
1%–49% (weakly positive)	NR	71 (75.5)	80 (35.6)	
≥50% (strongly positive)	NR	0 (0)	21 (9.33)	

Note.—Except where indicated, data are numbers of patients, with percentages in parentheses. A total of 319 patients with diverse demographics and tumor pathology were included in the analysis. Differences between distributions of participant characteristics in the training set and external test sets were tested using the Fisher exact test for categorical variables and an unpaired two-sided *t* test for age. NR = not reported, PD-L1 = programmed death ligand 1, TPS = tumor proportion score.

* Data are means ± SDs.

### Identification and Testing of CT Models

Of 17 published CT models identified from the literature review (Fig 3, Table 2), only three (18%) could be reconstructed from published information. For models 1–3, the training set was used only for preprocessing; external test set features were standardized using training set means and SDs. In contrast, model 3a coefficients were refitted in the training set, using the same features as model 3.

**Table 2:** Previously Published Studies Performed to Predict PD-L1 Expression in NSCLC

Author and Year	Tumor Stage	Sample Size	No. of Radiomic Features	PD-L1 TPS Threshold*	Model Architecture	Inclusion for Testing
Wen et al, 2017 (45)	NR	96	8	NR	Logistic regression	Model NR
Ruchalski et al, 2019 (46)	IV	97	22	1	Logistic regression with backward feature selection	Model NR
Yoon et al, 2020 (33)	IIIB–IVC	153	4	50	Logistic regression	Included for testing
Sun et al, 2020 (47)	I–IV	390	9	50	LASSO regression	Features could not be mapped to IBSI definitions
Tonneau et al, 2021 (48)	IV	299	NR	50	Neural network	Model NR
Bracci et al, 2021 (31)	IIIA–IV	72	2	50	Logistic regression	Included for testing
Jiang et al, 2021 (32)	Tis–III	125	9	1	Ridge regression with recursive feature elimination	Included for testing
Shiinoki et al, 2022 (49)	NR	161	NR	1 and 50	LASSO regression	Features not mapped to IBSI definitions
Wang et al, 2019 (50)	I–IV	126	NR	NR	Deep convolutional neural network	Features not mapped to IBSI definitions
Wen et al, 2021 (51)	III–IV	120	6	50	LASSO regression	Model coefficients NR
Wang et al, 2022 (52)	I–IV	3816	100 dimensional features	1 and 50	Deep convolutional neural network	Model NR
Wang et al, 2022 (53)	I–IV	1135	NR	1 and 50	Deep convolutional neural network	Model NR
Shi et al, 2022 (54)	I	839	9	50	LASSO regression	Model coefficients NR
Singh et al, 2022 (55)	IV	107	102	No threshold	Cox regression	Model NR
Chen et al, 2023 (56)	I–IV	194	15	1, 50, and 90	Elastic net regression	Features not mapped to IBSI definitions
Yolchuyeva et al, 2023 (57)	IV	385	NR	50	LASSO regression	Model NR
Fu et al, 2023 (58)	NR	43	1656	1	Neural network	Model NR

Note.—Three models were reported sufficiently for testing in the current study. IBSI = Image Biomarker Standardization Initiative, LASSO = least absolute selection and shrinkage operator, NR = not reported, NSCLC = non–small cell lung cancer, PD-L1 = programmed death ligand-1, Tis = tumor in situ, TPS = tumor proportion score.

* Data are percentages.

**Model 1**

Model 1 (31) was a logistic regression classifier of TPS_≥50%_, using two radiomic without image transforms. It was developed in 72 patients with NSCLC, 31 of whom had adenocarcinoma (≥ stage III), and 48 of whom were classified as PD-L1 positive. A second model discriminating TPS_≥1%_ could not be reconstructed due to an unidentifiable feature.

**Model 2**

Model 2 (32) was a logistic regression classifier for TPS_≥1%_, using nine radiomic features from multiple image transforms, developed in 91 patients with NSCLC (84 with adenocarcinoma, ≤ stage III); 27 were classified as PD-L1 positive.

**Model 3**

**Figure 4:**
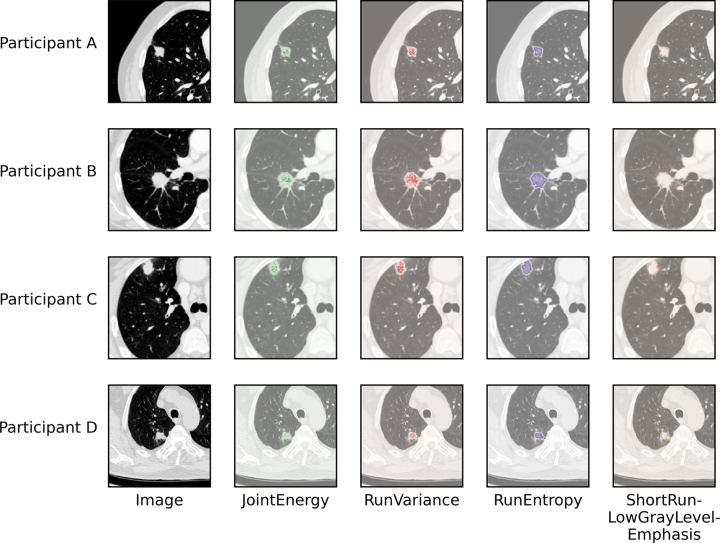
Voxel-level radiomic feature visualization in four patients. Participant A is a 54-year-old male with pT2 lung adenocarcinoma (programmed death ligand-1 [PD-L1] tumor proportion score [TPS], 0%). Participant B is a 70-year-old female with pT1 adenosquamous lung carcinoma (PD-L1 TPS, 0%). Participant C is a 68-year-old male with pT1 lung adenocarcinoma (PD-L1 TPS, 60%). Participant D is a 72-year-old male with pT2 lung adenocarcinoma (PD-L1 TPS, 55%). Images are, from left (original image) to right (corresponding features), CT image (black and white), Gray-level cooccurrence matrix JointEnergy (green), gray-level run-length matrix (GLRLM)_RunVariance (red), GLRLM_RunEntropy (purple), and GLRLM_ShortRunLowGrayLevelEmphasis (orange).

Model 3 (33) was a logistic regression model for TPS_≥50%_ and TPS_≥1%_, using four radiomic features (Joint Energy, Run Variance, Run Entropy, and Short Run Low Gray Level Emphasis) without image transforms, developed in 153 patients with NSCLC with adenocarcinoma (≥ stage III), 53 of whom were PD-L1 positive. Figure 4 shows these radiomic features at voxel-level in four patients from the external test set. Model 3 features (Joint Energy, Run Variance, Run Entropy, and Short Run Low Gray Level Emphasis) were selected for fitting to *CD274* mRNA expression (model 3a).

Table S3 provides the radiomics quality score for these three model studies.

### Discrimination of PD-L1 Protein Expression

**Figure 5:**
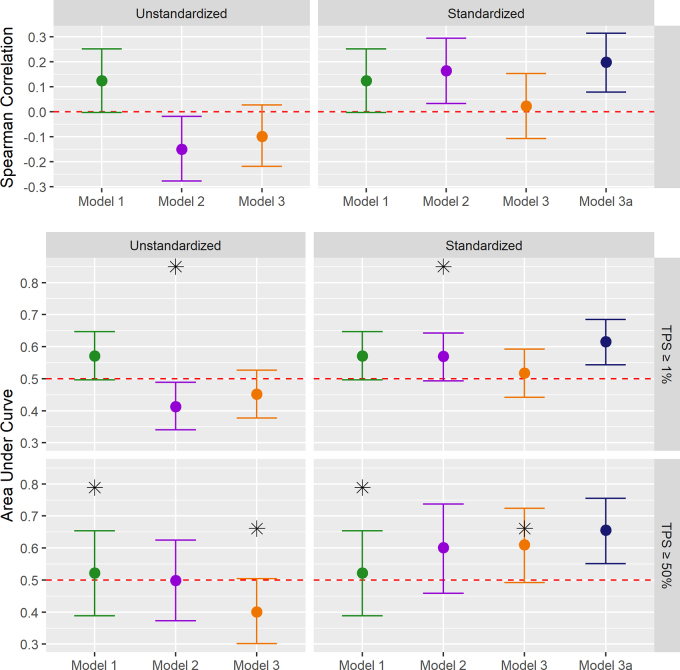
Dot and whisker plot shows discrimination performance of each model in the external test set. Model 1 (published by Bracci et al [31]) reported predictions correlated weakly with tumor proportion score (TPS) but underperformed published results. Model 2 (published by Jiang et al [32]) predictions discriminated TPS in standardized test data but underperformed published results. Model 3 (pubished by Yoon et al [33]) discriminated a TPS at a clinical threshold of at least 50% (TPS_≥50%_) with an area under the receiver operating characteristic curve (AUC) of 0.61 (95% CI: 0.49, 0.72) and model 3a (fitted to *CD274* messenger RNA expression, using model 3 features) with an AUC of 0.66 (95% CI: 0.55, 0.76). Top: Spearman correlation of model prediction with TPS. Dots represent point estimates of mean performance, error bars denote 95% CIs, and asterisks mark previously published performance results. Middle and bottom: AUCs for models classifying TPS at thresholds 1% (middle) and 50% (bottom), respectively. Unstandardized models were applied without any feature standardization. In standardized models, radiomic features were standardized using means and SDs estimated in the training set. Red dotted lines indicate the null values. TPS_≥1%_ = tumor proportion score at a clinical threshold of at least 1%.

Test performance is reported in Table 3 and Figure 5. Model 1 predictions correlated positively with PD-L1 expression quantile (*ρ* = 0.12 [95% CI: 0.00, 0.25]; *P* = .03 [vs null]), modestly discriminating the TPS_≥1%_ threshold (AUC, 0.57 [95% CI: 0.50, 0.65]; *P* = .03 [vs null]). However, discrimination at the TPS_≥50%_ threshold (AUC, 0.52 [95% CI: 0.39, 0.65]; *P* = .37 [vs null]) was lower than reported in the original study (AUC, 0.79; *P* < .001 [testing vs published]) (28). As model 1 was univariate, discrimination results were invariant to standardization.

**Table 3:** Model Discrimination Performance in Predicting PD-L1 TPS in the External Test Set

Model, Author, and Standardization	Spearman ρ	AUC for TPS_≥1%_	AUC for TPS_≥50%_
Model 1, Bracci et al (31)			
Unstandardized	0.12 (0.00, 0.25);* P* = .03 [vs null]	0.57 (0.50, 0.65); *P* = .03 [vs null]	0.52 (0.39, 0.65); *P* = .37 [vs null];*P* < .001 [vs published]
Standardized	0.12 (0.00, 0.25);* P* = .03 [vs null]	0.57 (0.50, 0.65); *P* = .03 [vs null]	0.52 (0.39, 0.65); *P* = .37 [vs null];*P* < .001 [vs published]
Model 2, Jiang et al (32)			
Unstandardized	−0.15 (−0.28, −0.01); *P* = .99 [vs null]	0.41 (0.34, 0.49); *P* = .99 [vs null];*P* < .001 [vs published]	0.50 (0.36, 0.62); *P* = .51 [vs null];
Standardized	0.16 (0.03, 0.30); *P* = .007 [vs null]	0.57 (0.49, 0.64); *P* = .04 [vs null]:*P* < .001 [vs published]	0.60 (0.46, 0.74); *P* = .08 [vs null];
Model 3, Yoon et al (33)			
Unstandardized	−0.10 (−0.22, 0.03); *P* = .93 [vs null]	0.45 (0.38, 0.52); *P* = .90 [vs null]	0.40 (0.30, 0.51); *P* = .97 [vs null];*P* < .001 [vs published]
Standardized	0.02 (−0.11, 0.16); *P* = .36 [vs null]	0.52 (0.44, 0.59); *P* = .32 [vs null]	0.61 (0.49, 0.72); *P* = .03 [vs null];*P* = .38 [vs published]
Model 3a (mRNA-fitted), standardized	0.20 (0.08, 0.32); *P* = .001 [vs null]	0.61 (0.55, 0.69); *P* = .001 [vs null]	0.66 (0.55, 0.76); *P* = .001 [vs null]

Note.—Except where indicated, data are areas under the receiver operating characteristic curve (AUCs), with 95% CIs in parentheses. Of three tested models, only model 3 achieved comparable test discrimination with published results. Model 3a achieved the best test discrimination overall. Spearman ρ denotes the correlation between model predictions and tumor proportion score (TPS) values. AUC was measured between model predictions and TPS at clinical thresholds of at least 1% (TPS_≥1%_) and 50% (TPS_≥50%_). For comparability, all *P* values are unadjusted. *P* values comparing test performance to null models (“vs null”) are one-sided and those comparing to published results are two-sided. For model 1, the AUC for TPS_≥50%_ was 0.79 (95% CI: 0.58, 1.00). For model 2, the AUC for TPS_≥1%_ was 0.85. For model 3, the AUC for TPS_≥50%_ was 0.66 (95% CI: 0.58, 0.74). mRNA = messenger RNA, PD-L1 = programmed death ligand-1.

Without standardization, models 2 and 3 were generally uninformative. With standardization, predictions improved. Model 2 predictions correlated with PD-L1 TPS ( *ρ* = 0.16 [95% CI: 0.03, 0.3]; *P* = .007 [vs null]), but discrimination of the TPS_≥1%_ threshold was lower (AUC, 0.57 [95% CI: 0.49, 0.64]; *P* = .04 [vs null]) than reported in the original study (AUC, 0.85; *P* < .001 [test vs published]) (32). Model 3 showed comparable discrimination of the TPS_≥50%_ threshold (AUC, 0.61 [95% CI: 0.49, 0.72]; *P* = .03 [vs null]) with that reported in the original study (AUC, 0.66; *P* = .38 [test vs published]) (33). However, there was no evidence that model 3 was predictive at the TPS_≥1%_ threshold (AUC, 0.52 [95% CI: 0.44, 0.59]; *P* = .32 [vs null]), and there was no evidence of a correlation between predictions and PD-L1 TPS quantiles ( *ρ* = 0.02 [95% CI: −0.11, 0.16]; *P* = .36).

The predictions of the *CD274* mRNA-fitted CT model (model 3a) correlated with PD-L1 TPS ( ρ = 0.20 [95% CI: 0.08, 0.32]; *P* = .001 [vs null]), discriminating both the TPS_≥1%_ threshold (AUC, 0.61 [95% CI: 0.55, 0.69]; *P* = .001 [vs null]) and the TPS_≥50%_ threshold (AUC, 0.66 [95% CI: 0.55, 0.76]; *P* = .001 [vs null]).

### Model Calibration

Model calibration results are presented in Table S4. Calibration of model 2 could not be assessed as the model intercept and decision threshold were not published. Without standardization, neither model 1 nor model 3 were calibrated sufficiently to yield useful predictions. With standardization, model 1 was not sufficiently specific for clinical application (specificity, 5% [95% CI: 2, 8]). With standardization, model 3 achieved a modest specificity (78% [95% CI: 72, 84]) and low sensitivity (33% [95% CI: 13, 55]).

### Post Hoc Analyses

Correlations and partial correlations among radiomic features, tumor volume, *CD274*, and PD-L1 quantiles are provided in Table S5. There was no evidence of a correlation between tumor volume and PD-L1 TPS quantile (*r* = .06 [95% CI: −0.07, 0.21]; *P* = .44 [vs null]). Gray-level run-length matrix run variance demonstrated sign-consistent correlations with *CD274* quantile in the training set (*r* = −.13 [95% CI: −0.25, 0.02]; *P* = .07 [vs null]) and PD-L1 quantile in the external test set (*r* = −.19 [95% CI: −0.29, −0.09]; *P* < .001 [vs null]). The partial correlation between the gray-level run-length matrix run variance and PD-L1 TPS quantile remained negative given tumor volume and the other radiomic predictors (partial correlation, −0.15 [95% CI: −0.25, −0.05]; *P* = .005 [vs null]). Model 3 predictions correlated marginally with voxel width (ρ = −0.15 [95% CI: −0.27, −0.04]; *P* = .02 [vs null]) and model 1 predictions correlated marginally with tube current (ρ = 0.14 [95% CI: 0.01, 0.25]; *P* = .03 [vs null]). Otherwise, there was no evidence of a correlation among model predictions and tube current, peak voltage, or voxel width (Table S6).

Deep learning segmentation performance is detailed in Appendix S1. With standardization, models attained similar test discrimination performance on radiomic features extracted with deep learning segmentation masks (Table S7). Model 3 maintained performance at the TPS_≥50%_ threshold (AUC, 0.63 [95% CI: 0.48, 0.76]; *P* = .035 [vs null]; *P* = .27 [vs all samples]), as did model 3a (AUC, 0.67 [95% CI: 0.53, 0.8]; *P* = .007 [vs null]; *P* = .56 [vs all samples]).

With standardization, models attained similar test discrimination performance when tumors with a volume of less than 5 mL were excluded (Table S8). Model 3 maintained performance at the TPS_≥50%_ threshold (AUC, 0.60 [95% CI: 0.49, 0.72]; *P* = .04 [vs null]; *P* = .45 [vs all samples]), as did model 3a (AUC, 0.66 [95% CI: 0.55, 0.77]; *P* = .002 [vs null]; *P* = .51 [vs all samples]).

## Discussion

Determining programmed cell death ligand-1 (PD-L1) status in operable non–small cell lung cancer (NSCLC) may optimize neoadjuvant (13) and adjuvant treatment with immunotherapy (7,34). Both fluorine 18 fluorodeoxyglucose PET (35–38) and CT features (17) have been associated with PD-L1 expression, although imaging use in this setting remains limited. The clinical utility of imaging models hinges on reproducibility and biologic explainability. Our study tested previously published CT PD-L1 models in an external test set of 225 patients with stage IIB–IIIB NSCLC undergoing surgical resection, where 48% of tumors were PD-L1 positive and 8% were strongly positive. Most models lacked sufficient reporting for replication; only three of 17 could be reconstructed. Model 1 achieved an area under the receiver operating characteristic curve (AUC) of 0.52 for a TPS at a clinical threshold of at least 50% (TPS_≥50%_) (*P* = .37 vs the null model; *P* < .001 [vs published]), model 2 an AUC of 0.57 for a TPS at a clinical threshold of at least 1% (*P* = .04 vs null; *P* < .001 [vs published]), and model 3 an AUC of 0.61 for TPS_≥50%_ (*P* = .03 vs null; *P* = .38 [vs published]). Only model 3 matched its original reported performance. A model refitted to *CD274* messenger RNA (model 3a) achieved the best discrimination (AUC of 0.66 for TPS_≥50%_;* P* = .001 [vs null]).

Several CT radiomics studies have retested models from the same group, often showing reduced performance on validation (39–42). Testing by external investigators is preferable as it assesses sufficiency of reported information for model reconstruction. Guevorguian and colleagues (43) tested a radiomic model developed by an external group for oropharyngeal carcinoma prognostication, discriminating overall survival as effectively as previously reported (44). Reproducibility is a core tenet of an image model. Studies should provide sufficient information on essential items for the independent reconstruction of models, including extraction, normalization, or transformation parameters. For example, missing transformation parameters (eg, rescaling factor, offset factor, logarithmic base) prevent accurate reconstruction and invalidate coefficient magnitudes.

In the three published models tested for discrimination, only model 3 matched published discrimination results; models 1 and 2 underperformed. Calibration is important for clinical implementation. Although some models showed marginal discrimination, effective calibration was not possible due to missing details such as radiomic feature standardization. Consequently, reported decision thresholds were uninformative. This overlooked issue needs improvement to enable independent model testing and support clinical implementation.

A strength of our study is its design, which evaluated the performance of models 1–3 without refitting. Where *CD274* mRNA expression was modeled, features were limited to those previously proposed, constraining dimensionality and the risk of overfitting.

There are limitations to our retrospective analysis. Two-thirds of identified models were untestable due to insufficient reporting. Although this result reflects the real-world clinical applicability of published models, it remains possible that some untested models might have performed if they had been reconstructed. To approximate real-world performance, models were tested in a multicenter dataset with heterogeneous imaging, which may have reduced performance compared with homogeneous data. Tumor stages and populations analyzed in the modeling studies may differ from those evaluated in the external test set. Prospective, multicenter validation remains a priority.

With increasing perioperative use of immune checkpoint inhibitors in non–small cell lung cancer, better programmed cell death ligand-1 (PD-L1) assessment of early-stage disease is needed. In conclusion, in independent testing, CT models discriminated PD-L1 expression at clinically relevant thresholds but underperformed reported results. Poor reporting for model reconstruction, lack of biologic explainability, and inadequate calibration hinder clinical implementation—issues future studies must address. Future work will aim to assess the generalizability of multiomics and multimodel PD-L1 prediction models, which present opportunities and further challenges.

## Supplemental Files

Appendix S1, Tables S1-S8, Figure S1

Conflicts of Interest
