# Supplementary material for: Independent Testing of Published CT Models for PD-L1 Status in Non–Small Cell Lung Cancer
Source: Radiology. 2026 Mar 31;318(3):e241962. doi: 10.1148/radiol.241962 (PMC13044353; doi:10.1148/radiol.241962)

Supplemental materials undergo peer review but are not copyedited, and may include typographical errors.

©RSNA, 2026
10.1148/radiol.241962

Appendix S1

Literature Search Strings

Pubmed Query

“((radiomic*[Title] OR radiogenomic*[Title] OR quantitative [Title] OR texture*[Title])

AND (computed tomography[Title/Abstract] OR ct[Title/Abstract])

AND (pd-l1[Title] OR pdl1[Title] OR programmed death ligand 1[Title] OR programmed cell death protein[Title] OR programmed cell death ligand[Title])

AND (nsclc[Title/Abstract] OR lung [Title/Abstract]))

AND ((humans[Filter])AND (english[Filter]))

2017/01:2023/08 [edat]“

Ovid Query

“((radiomic* OR radiogenomic* OR quantitative OR texture*).ti.

AND (computed tomography OR ct).ti,ab.

AND (pd-l1 OR pdl1 OR programmed death ligand 1 OR programmed cell death protein OR programmed cell death ligand).ti.

AND (nsclc OR lung).ti,ab.)

Filters: English Language, Humans, Publication Year 2017-2023”

Software Libraries

Supplemental materials undergo peer review but are not copyedited, and may include typographical errors.

Image processing was performed in python version 3.7.10.

Deep learning segmentation model

A deep learning segmentation (DLS) model was trained for tumor segmentation on the public dataset. Axial image patches of dimension 128*128 were sampled randomly with centre-points in the tumor volume. A random patient-disjoint 80:20 partition of the public dataset was used for model training and internal validation. Images were augmented by the addition of random Gaussian noise with mean zero and standard deviation 0.001.

A 2d attention u-net architecture was implemented with five convolutional blocks (filter depth: 16, 32, 64, 128, 256) and five deconvolutional blocks (filter depth: 128, 64, 32, 16). Convolutional blocks consisted of the following layers: convolution/deconvolution, spatial dropout, batch normalisation, convolution/deconvolution, downsampling/upsampling. Spatial dropout was applied with a rate of 0.25. Hidden activations were rectified linear units and output activations were logistic sigmoid. Segmentation dice loss was minimised with the Adam optimiser using initial learning rate of 0.001, with decay factor of 0.5 following 10 epoch loss plateau. Training continued for 50 epochs.

In deployment on the test dataset, the DLS model was deployed as a sliding window (step size: 50 voxels) over the tumor volume, defined around the manually annotated tumor mask with a margin of 96 voxels in each direction in the axial plane. Overlapping predictions were combined by averaging prediction logits for each voxel before applying a logit threshold of 0 to define positive voxels.

Automated segmentation results

Supplemental materials undergo peer review but are not copyedited, and may include typographical errors.

Learning curves for segmentation model performance on training and validation partitions are training are provided in **Figure S1.** In the final training epoch, the model achieved training and internal validation dice scores of 0.85 and 0.73 respectively in the public dataset. Segmentation performance was lower in the test dataset (Dice score: 0.61 [mean] ± 0.26 [sd], Precision: 0.61 ± 0.22; Recall: 0.72 ± 0.35). All images in which the automated segmentation model achieved a dice score < 0.5 (n=55) were reviewed by a radiologist. The following (non-mutually exclusive) tumoral/peri-tumoral conditions were identified as complicating clinical factors: consolidation: n=10; atelectasis/collapse: n=25; pleural disease: n=2; severe parenchymal disease: n=5; effusion/hydropneumothorax: n=2; cavitation: n=7; motion artefact: n=2.

Supplemental References

1. Cui R, Yang Z, Liu L. What does radiomics do in PD-L1 blockade therapy of NSCLC patients? Thorac Cancer 2022;13(19):2669-2680.
2. Wen Q, Meng X, Zhu J, Yin Y, Yu J. CT radiomics features for evaluation of PD-L1, CD8+TILs and Foxp3+TILs expression status in patients with stage I NSCLC. *Med Phys*. 2017;44:2784.
3. Yoon J, Suh YJ, Han K, Cho H, Lee HJ, Hur J, et al. Utility of CT radiomics for prediction of PD-L1 expression in advanced lung adenocarcinomas. *Thorac Cancer*. 2020;11:993–1004.
4. Sun Z, Hu S, Ge Y, Wang J, Duan S, Song J, et al. Radiomics study for predicting the expression of PD-L1 in non-small cell lung cancer based on CT images and clinicopathologic features. *J X-ray Sci Technol*. 2020;28:449–59.
5. Bracci S, Dolciami M, Trobiani C, Izzo A, Pernazza A, D'Amati G, et al. Quantitative CT texture analysis in predicting PD-L1 expression in locally advanced or metastatic NSCLC patients. *Radiol Med*. 2021;126:1425–33.
6. Jiang Z, Dong Y, Yang L, Lv Y, Dong S, Yuan S, et al. CT-based hand-crafted radiomic signatures can predict PD-L1 expression levels in non-small cell lung cancer: a two-center study. *J Digit Imaging*. 2021;34:1073–85.
7. Shiinoki T et al. Predicting PD-L1 expression level in non-small cell lung cancer on computed tomography using machine learning. *Med Phys*. 2021;48:e190.
8. Wang C, Ma J, Shao J, Zhang S, Liu Z, Yu Y, et al. Predicting EGFR and PD-L1 status in NSCLC patients using multitask AI system based on CT images. *Front Immunol*. 2022;13:813072.
9. Wen Q, Yang Z, Dai HH, Feng A, Li Q. Radiomics study for predicting the expression of PD-L1 and tumor mutation burden in non-small cell lung cancer

Supplemental materials undergo peer review but are not copyedited, and may include typographical errors.

based on CT images and Clinicopathological features. *Front Oncol*. 2021;11:620246.

10. Wang C, Xu X, Shao J, Zhou K, Zhao K, He Y, et al. Deep learning to predict EGFR mutation and PD-L1 expression status in non-small-cell lung cancer on computed tomography images. *J Oncol*. 2021;2021:1–11.
11. Wang C, Ma J, Shao J, Zhang S, Li J, Yan J, et al. Non-invasive measurement using deep learning algorithm based on multi-source features fusion to predict PD-L1 expression and survival in NSCLC. *Front Immunol*. 2022;13:828560.
12. Chen M, Lu H, Copley SJ, Han Y, Logan A, Viola P, Cortellini A, Pinato DJ, Power D, Aboagye EO. A Novel Radiogenomics Biomarker for Predicting Treatment Response and Pneumotoxicity From Programmed Cell Death Protein or Ligand-1 Inhibition Immunotherapy in NSCLC. *J Thorac Oncol* 2023;18(6):718-730.

Supplemental materials undergo peer review but are not copyedited, and may include typographical errors.

Table S1. Summary of CT acquisition parameters.

**Variable**	**Value**	**Public** *(n, %)*	**Test** *(n, %)*	**All** *(n, %)*
Scanner Manufacturer	GE MEDICAL SYSTEMS	76 (81)	31 (14)	107 (34)
	PHILIPS	2 (2)	89 (40)	91 (29)
	SIEMENS	13 (14)	69 (31)	82 (26)
	TOSHIBA	1 (1)	36 (16)	37 (12)
	UNKNOWN	2 (2)	0 (0)	2 (1)
Reconstruction Kernel	B	0 (0)	66 (29)	66 (21)
	B20f	0 (0)	29 (13)	29 (9)
	FC03	0 (0)	21 (9)	21 (7)
	lung	47 (50)	0 (0)	47 (15)
	other	47 (50)	109 (48)	156 (49)
Tube Current (mA)	(0,200]	18 (19)	100 (44)	118 (37)
	(200,500]	48 (51)	95 (42)	143 (45)
	(500,1000]	26 (28)	14 (6)	40 (13)
	unknown	2 (2)	16 (7)	18 (6)
Peak Voltage (kVp)	90	0 (0)	1 (0)	1 (0)
	100	3 (3)	33 (15)	36 (11)
	110	0 (0)	6 (3)	6 (2)
	120	91 (97)	184 (82)	275 (86)
	140	0 (0)	1 (0)	1 (0)
Voxel Width (mm)	(0.5,0.7]	17 (18)	72 (32)	89 (28)

Supplemental materials undergo peer review but are not copyedited, and may include typographical errors.

	(0.7,0.9]	65 (69)	141 (63)	206 (65)
	(0.9,1]	12 (13)	9 (4)	21 (7)
	unknown	0 (0)	3 (1)	3 (1)

Supplemental materials undergo peer review but are not copyedited, and may include typographical errors.

Table S2. Summary of model features. Radiomic parameters were extracted following either the proposing study, or Ligero et al. Feature Mean and SD are the parameters by which radiomic features were standardized, which were estimated from the public dataset.

Model	Radiomic Feature	Feature Mean	Feature SD	β	Radiomic extraction parameters
1	Intercept	NA	NA	-1.192	Ligero
	Original_firstorder_Skewness	0.1086	1.1817	-0.937	Ligero
	Original_GLSZM LowGrayLevelZone Emphasis	0.0374	0.0759	-11259862	Ligero
2	Intercept	NA	NA	0	Jiang
	log.sigma.4.0.mm.3D GLSZM SmallAreaLow GrayLevelEmphasis	0.0058	0.0141	-1.09552856	Jiang
	Wavelet.LHL GLCM_ClusterShade	-203.494	444.0348	-0.86632614	Jiang
	Wavelet.LHH firstorder_Kurtosis	5.3918	2.016	0.58220868	Jiang
	wavelet.LHH_glcm_Idn	0.9145	0.0383	0.97471849	Jiang
	Wavelet.HLL firstorder RootMeanSquared	980.6587	16.6257	-0.77145334	Jiang
	Wavelet.HLL NGTDM_Complexity	1135.3104	1254.6204	-0.9226715	Jiang
	Wavelet.HHH firstorder_Median	0.1285	0.6391	0.84877258	Jiang
	Wavelet.HHH firstorder_Skewness	-0.0399	0.1654	0.52749997	Jiang
	Wavelet.HHH GLSZM_SizeZoneNon	0.4342	0.0717	0.77429202	Jiang

Supplemental materials undergo peer review but are not copyedited, and may include typographical errors.

	UniformityNormalized				
3	Intercept	NA	NA	-1.59423	Ligero
	Original_GLCM_ JointEnergy	0.0213	0.0293	-8.49568	Ligero
	Original_GLRLM_ RunVariance	0.2253	0.3428	3.58597	Ligero
	Original_ GLRLM _RunEntropy	4.1537	0.2959	-5.01416	Ligero
	Original_ GLRLM ShortRunLow GrayLevelEmphasis	0.0208	0.0428	0.05253	Ligero
3a	Intercept	NA	NA	0.5053	Ligero
	Original_GLCM JointEnergy	0.0213	0.0293	0.0208	Ligero
	Original_GLRLM RunVariance	0.2253	0.3428	-0.0457	Ligero
	Original_GLRLM RunEntropy	4.1537	0.2959	0.0028	Ligero
	Original_GLRLM ShortRunLowGrayLevel Emphasis	0.0208	0.0428	-0.0044	Ligero

Supplemental materials undergo peer review but are not copyedited, and may include typographical errors.

Table S3. Radiomics quality scores of reconstructed models

Radiomics Quality Score item	Model 1 Bracci	Model 2 Jiang	Model 3 Yoon	Model 3a (current study)
Image protocol quality	Image protocols described	Image protocols described	Image protocols described	Varied imaging protocols employed
Multiple segmentations	Yes	Yes	Yes	Yes
Phantom study on all scanners	No	No	No	No
Imaging at multiple time points	Yes	No	No	No
Feature reduction	Yes	Yes	Yes	Yes
Multivariable analysis with non-radiomics features	No	No	Yes	No
Detect and discuss biological correlates	Yes	Yes	Yes	Yes
Cut-off analyses	Yes	No	Yes	Yes
Discrimination statistics	Discrimination & significance reported. Resampling also applied.	Discrimination & significance reported. Resampling also applied	Discrimination & significance reported. Resampling also applied.	Discrimination & significance reported. Resampling also applied.
Calibration statistics	Calibration & significance reported. Resampling also applied.	Calibration & significance reported.	Calibration & significance reported. Resampling also applied.	Calibration & significance reported. Resampling also applied.
Prospective study	No	No	No	No
Validation	Validation based on a	Validation based on a	Validation based on a	Validation based on

Supplemental materials undergo peer review but are not copyedited, and may include typographical errors.

	dataset from the same institution.	dataset from a different institution.	dataset from the same institution.	three or more datasets from distinct institutions. Validates previously proposed radiomic signature.
Gold standard comparison	Not applicable	Not applicable	Not applicable	Not applicable
Clinical utility	Yes	Yes	Yes	Yes
Cost effectiveness	No	No	No	No
Open science and data	No	No	No	Code is open sourced. Radiomic features & representative ROIs are open source
Total score	16	15	16	18

Supplemental materials undergo peer review but are not copyedited, and may include typographical errors.

Table S4. Model calibration performance in the test dataset.

Model	Harmonization	Accuracy *(95% CI)*	Sensitivity *(95% CI)*	Specificity *(95% CI)*	Precision	F1 Score
Model 1	Unstandardized	91% [86%, 95%]	0% [0%, 0%]	100% [100%, 100%]	0 [0, 0]	0 [0, 0]
Model 1	Standardized	13% [9%, 18%]	96% [84%, 100%]	5% [2%, 8%]	0.09 [0.06, 0.14]	0.17 [0.1, 0.24]
Model 3	Unstandardized	91% [86%, 95%]	0% [0%, 0%]	100% [100%, 100%]	0 [0, 0]	0 [0, 0]
Model 3	Standardized	74% [68%, 80%]	33% [13%, 55%]	78% [72%, 84%]	0.14 [0.05, 0.24]	0.19 [0.07, 0.32]

Supplemental materials undergo peer review but are not copyedited, and may include typographical errors.

Table S5. Feature correlation analysis. Correlation of Model 3 features and tumor volume with CD274 quantile in the public dataset and PD-L1 tumor proportion score quantile in the external test dataset. Partial correlations of each feature were also computed, given the other four features. 95% confidence intervals and P values were estimated by bootstrapping with replacement. P-values are two-sided and unadjusted.

**Dataset**	**Public Dataset**	**Test Dataset**	**Public Dataset**	**Test Dataset**
**Gene Association**	**CD274 Correlation** *(95% CI)*	**PD-L1 Correlation** *(95% CI)*	**CD274 Partial Correlation** *(95% CI)*	**PD-L1 Partial Correlation** *(95% CI)*
**Radiomic Feature**				
GLRLM _RunEntropy	0 [-0.18, 0.19], *P*=.99 [vs. null]	-0.1 [-0.23, 0.02], *P*=.11 [vs. null]	-0.01 [-0.2, 0.17], *P*=.91 [vs. null]	-0.05 [-0.18, 0.07], *P*=.41 [vs. null]
GLRLM _RunVariance	-0.13 [-0.25, 0.02], *P*=.07 [vs. null]	-0.19 [-0.29, -0.09], *P*<.001 [vs. null]	-0.11 [-0.25, 0.08], *P*=.22 [vs. null]	-0.15 [-0.25, -0.05], *P*=.005 [vs. null]
GLRLM_ShortRun-LowGrayLevelEmphasis	0 [-0.17, 0.18], *P*>.99 [vs. null]	-0.09 [-0.2, 0.01], *P*=.10 [vs. null]	-0.02 [-0.2, 0.16], *P*=.77 [vs. null]	-0.12 [-0.25, -0.01], *P*=.03 [vs. null]
GLCM_JointEnergy	-0.02 [-0.22, 0.12], *P*=.92 [vs. null]	-0.11 [-0.23, 0.01], *P*=.08 [vs. null]	0.04 [-0.13, 0.16], *P*=.50 [vs. null]	0.06 [-0.05, 0.18], *P*=.28 [vs. null]
Shape_VoxelVolume	-0.09 [-0.26, 0.15], *P*=.36 [vs. null]	0.06 [-0.07, 0.21], *P*=.44 [vs. null]	-0.08 [-0.26, 0.18], *P*=.43 [vs. null]	0.01 [-0.13, 0.17], *P*=.88 [vs. null]

Supplemental materials undergo peer review but are not copyedited, and may include typographical errors.

Table S6. Model sensitivity analysis. Spearman correlations between model predictions and image acquisition parameters in the test cohort are shown. 95% confidence intervals and P values were estimated by bootstrapping with replacement. P values are two-sided and unadjusted.

**Image Acquisition Parameter**	**Tube Current Correlation** *(95% CI, P value)*	**Peak Voltage Correlation** *(95% CI, P value)*	**Voxel width Correlation** *(95% CI, P value)*
**Model**			
Model 1	0.14 [0.01,0.25], *P*=.03 [vs. null]	-0.05 [-0.19,0.07], *P*=.40 [vs. null]	-0.02 [-0.15,0.1], *P*=.71 [vs. null]
Model 2	0.07 [-0.07,0.2], *P*=.33 [vs. null]	0.03 [-0.09,0.16], *P*=.58 [vs. null]	-0.05 [-0.16,0.07], *P*=.47 [vs. null]
Model 3	0.02 [-0.12,0.15], *P*=.81 [vs. null]	0.04 [-0.1,0.18], *P*=.53 [vs. null]	-0.15 [-0.27,-0.04], *P*=.02 [vs. null]
Model 3a	0.09 [-0.04,0.21], *P*=.14 [vs. null]	0.05 [-0.07,0.18], *P*=.47 [vs. null]	-0.02 [-0.14,0.11], *P*=.80 [vs. null]

Supplemental materials undergo peer review but are not copyedited, and may include typographical errors.

Table S7. Model discrimination performance in test dataset, using features extracted from automated segmentation masks. Results include N=170 patients for whom the segmentation model achieved a dice score > 0.5 ($TPS_{\geq 1\%}$: n=77; $TPS_{\geq 50\%}$: n=15). Spearman rho denotes correlation between model predictions and PD-L1 tumor proportion score (TPS) values. Area under the curve (AUC) was measured between model predictions and TPS positivity thresholds of ≥1% and 50%. For comparability, all P-values are unadjusted. P values comparing performance versus null models are one-sided and those comparing performance versus all samples are two-sided.

**Model**	**Harmonization**	**Spearman Rho** *(95% CI)*	**AUC $TPS_{\geq 1\%}$** *(95% CI)*	**AUC $TPS_{\geq 50\%}$** *(95% CI)*
Model 1	Standardized	-0.03 [-0.18, 0.14] *P*=.66 [vs. null], *P*=.07 [vs. all samples]	0.47 [0.39, 0.55] *P*=.77 [vs. null], *P*=.051 [vs. all samples]	0.51 [0.33, 0.7] *P*=.46 [vs. null], *P*=.44 [vs. all samples]
Model 2	Standardized	0.19 [0.05, 0.33] *P*=.006 [vs. null], *P*=.61 [vs. all samples]	0.59 [0.5, 0.67] *P*=.025 [vs. null], *P*=.63 [vs. all samples]	0.53 [0.37, 0.69] *P*=.35 [vs. null], *P*=.27 [vs. all samples]
Model 3	Standardized	0.01 [-0.15, 0.16] *P*=.45 [vs. null], *P*=.46 [vs. all samples]	0.50 [0.41, 0.59] *P*=.50 [vs. null], *P*=.39 [vs. all samples]	0.63 [0.48, 0.76] *P*=.035 [vs. null], *P*=.56 [vs. all samples]
Model 3a	Standardized	0.22 [0.07, 0.36] *P*=.002 [vs. null], *P*=.59 [vs. all samples]	0.62 [0.54, 0.7] *P*=.003 [vs. null], *P*=.55 [vs. all samples]	0.67 [0.53, 0.8] *P*=.007 [vs. null], *P*=.56 [vs. all samples]

Supplemental materials undergo peer review but are not copyedited, and may include typographical errors.

Table S8. Model discrimination performance in test dataset, excluding cases with tumor volume < 5ml. Results include N=220 cases ($TPS_{\geq 1\%}$: n=99; $TPS_{\geq 50\%}$: n=19). Spearman rho denotes correlation between model predictions and PD-L1 tumor proportion score (TPS) values. Area under the curve (AUC) was measured between model predictions and TPS positivity thresholds of ≥1% and 50%. For comparability, all P-values are unadjusted. P values comparing performance versus null models are one-sided and those comparing performance versus all samples are two-sided.

**Model**	**Harmonization**	**Spearman Rho** *(95% CI)*	**AUC $TPS_{\geq 1\%}$** *(95% CI)*	**AUC $TPS_{\geq 50\%}$** *(95% CI)*
Model 1	Standardized	0.13 [0.01, 0.25] *P*=.03 [vs. null], *P*=.55 [vs. all samples]	0.57 [0.49, 0.64] *P*=.03 [vs. null], *P*=.51 [vs. all samples]	0.54 [0.41, 0.67] *P*=.28 [vs. null], *P*=.57 [vs. all samples]
Model 2	Standardized	0.16 [0.03, 0.29] *P*=.007 [vs. null], *P*=.51 [vs. all samples]	0.57 [0.48, 0.64] *P*=.04 [vs. null], *P*=.47 [vs. all samples]	0.61 [0.46, 0.76] *P*=.07 [vs. null], *P*=.51 [vs. all samples]
Model 3	Standardized	0.02 [-0.11, 0.15] *P*=.37 [vs. null], *P*=.48 [vs. all samples]	0.52 [0.44, 0.59] *P*=.30 [vs. null], *P*=.52 [vs. all samples]	0.60 [0.49, 0.72] *P*=.04 [vs. null], *P*=.45 [vs. all samples]
Model 3a	Standardized	0.21 [0.09, 0.33] *P*=.001 [vs. null], *P*=.57 [vs. all samples]	0.62 [0.55, 0.69] *P*<.001 [vs. null], *P*=.58 [vs. all samples]	0.66 [0.55, 0.77] *P*=.002 [vs. null], *P*=.51 [vs. all samples]

Supplemental materials undergo peer review but are not copyedited, and may include typographical errors.

Figure S1. Learning curve for the automated segmentation model over 50 epochs training. Training and internal validation partitions were a patient-disjoint 80:20 split of the public dataset.

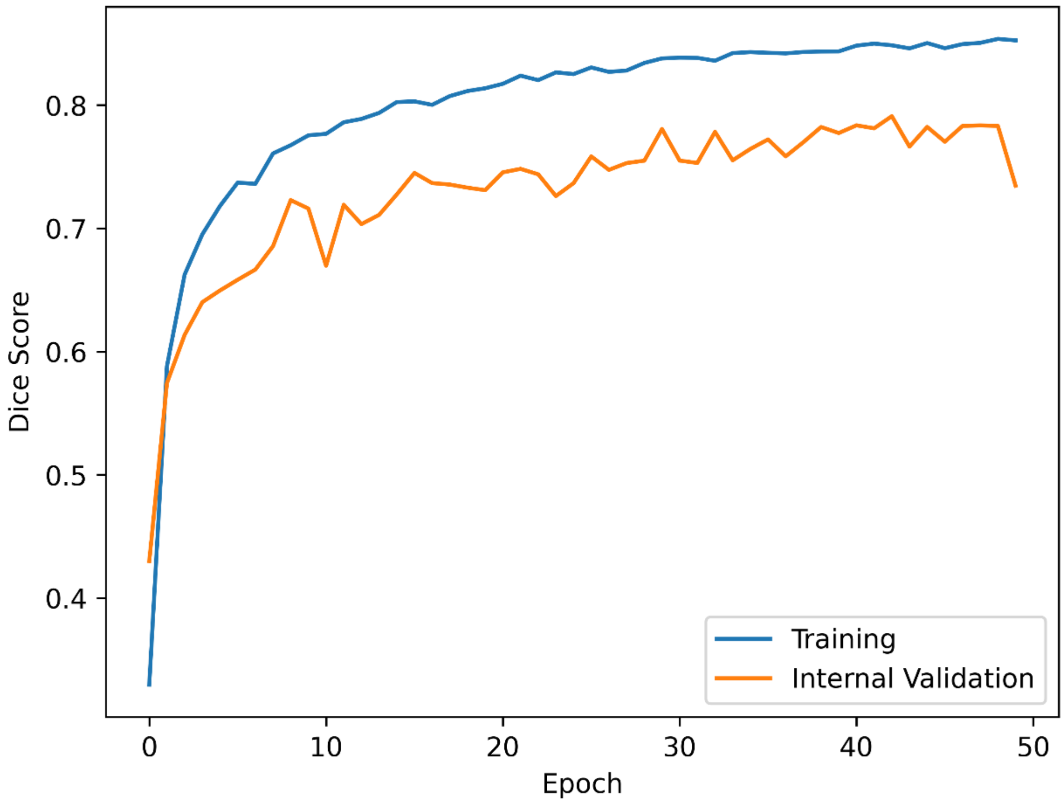

Supplement: Appendix S1, Tables S1-S8, Figure S1 [file ry241962supp.pdf]
